# Perifosine and vitamin D combination induces apoptotic and non-apoptotic cell death in endometrial cancer cells

**DOI:** 10.17179/excli2019-1834

**Published:** 2020-05-04

**Authors:** Meryem Ilkay Karagul, Savas Aktas, Sakir Necat Yilmaz, Derya Yetkin, Havva Didem Celikcan, Ozge Selin Cevik

**Affiliations:** 1 Department of Histology and Embryology, Faculty of Medicine, Mersin University, Mersin, Turkey; 2 Advanced Technology of Education, Research and Application Center, Mersin University, Mersin, Turkey; 3 Department of Biostatistics and Medical Informatics, Faculty of Medicine, Mersin University, Mersin, Turkey; 4 Department of Physiology, Faculty of Medicine, Mersin University, Mersin, Turkey

**Keywords:** endometrial cancer, proliferation, apoptosis, BCL2, BAX, P53

## Abstract

Endometrial cancer is the most common cancer of the female reproductive system. Combination treatment with specific agents has been widely used as a targeted therapy for cancer. In this study, we aimed to investigate the anti-proliferative and apoptotic effects of varying concentrations of perifosine and vitamin D on the human endometrial cancer cell line (HEC-1A). HEC-1A cells were exposed to perifosine (10 μM, 30 μM), vitamin D (50 nM, 200 nM) and combinations of both for 48 h and 72 h. Monitoring of cell proliferation in a time-dependent manner was performed with the xCELLigence RTCA DP system. The levels of BCL2, BAX and P53 mRNA expression were examined using RT-qPCR. Apoptosis was determined using Annexin V, which were followed by flow cytometry analysis. Ultra-structural morphology of cells was analyzed by transmission electron microscopy (TEM) for 72 h. The anti-proliferative and apoptotic effects of the perifosine+vitamin D combination (30 μM + 200 nM at 48 h and 10 μM + 200 nM at 72 h) on HEC-1A cells were higher than in perifosine and vitamin D alone. It was observed that perifosine has increased the expression of BAX mRNA in HEC-1A cells in a dose-dependent manner. While perifosine+vitamin D combinations increased P53 mRNA expression in HEC-1A cells we did not find any significant change in BCL2, BAX mRNA expression levels. In TEM examinations of HEC-1A cells, perifosine appeared to lead autophagic cell death, whereas vitamin D caused paraptosis-like cell death and combination of perifosine+vitamin D caused apoptotic and non-apoptotic (paraptotic, autophagic and necrotic) cell death. Therefore, it is considered that the combination of both drugs in the treatment of endometrial cancer might be an alternative and effective treatment option through activating the apoptotic and non-apoptotic cell death mechanisms in cancer cells.

## Introduction

Endometrial carcinoma is the most common cancer of the female reproductive system that presents as two different clinicopathologic conditions (an estrogen-dependent type and an estrogen-independent type) (Crosbie et al., 2010[7]). It is a cancer of postmenopausal women; worldwide, 91 % of cases take place in women aged 50 and older (Parkin et al., 2005[28]). 

Perifosine is a synthetic alkylphospholipid, which has demonstrated anti-tumor activity by inhibition of protein kinase B (AKT) and the mammalian target of rapamycin (mTOR) axis (Engel et al., 2008[9]). Phosphatidylinositol-3 kinase (PI3K)/AKT pathway activation has been associated with malignant transformation and apoptotic resistance (Hennessy et al., 2005[15]; Vivanco and Sawyers, 2002[37]). Downstream signaling of PI3K/AKT is the mTOR pathway, which plays a critical role in the regulation of proliferation, survival, and autophagy (Engel et al., 2008[9]). Perifosine has indicated significant anti-proliferative activity in several human tumor model systems and has recently been tested in clinical phase II studies in breast, prostate, head and neck cancers (Argiris et al., 2006[1]; Hideshima et al., 2006[17]; Leighl et al., 2008[22]). 

Vitamin D is a hormone that can be acquired from the diet or produced endogenously by a series of reactions that culminate in the most active metabolite of 1,25-dihydroxyvitamin D_3_ (1,25(OH)_2_D_3_ or calcitriol) (Banerjee and Chatterjee, 2003[3]; Rao et al., 2002[32]). The active metabolite of vitamin D has anti-proliferative and pro-apoptotic features (Banerjee and Chatterjee, 2003[3]). 1,25(OH)_2_D_3_ biological activities require binding to the cytosolic vitamin D receptor (VDR) (Mariani et al., 2012[25]). It has been shown that human cycling endometrium expresses the VDR, and a few studies have demonstrated the functional role of 1,25(OH)_2_D_3 _in female reproduction system (Mariani et al., 2012[25]; Shand et al., 2010[34]; Vigano et al., 2006[36]). 1,25(OH)_2_D_3 _shows anti-proliferative effects in a variety of cancer cell types including cell lines derived from prostate, ovarian, endometrial and breast (Axanova et al., 2010[2]; Kasiappan et al., 2014[19]). The anti-cancer mechanisms of 1,25(OH)_2_D_3_ contain induction of cell cycle arrest, promotion of differentiation, inhibition of invasive and migratory potential of cancer cells (Krishnan et al., 2003[21]).

Apoptosis plays a part in the development and maintenance of homeostasis in a variety of tissues including the female reproductive system. It is an important regulator in the reconstruction of the endometrium during the menstrual cycle. Extracellular signals, as well as an intracellular autonomous genetic program, regulate apoptosis (Argiris et al., 2006[1]). Inactivation of tumor-suppressor genes (TSG) is a molecular target for the development of neoplasia. P53 is one of the TSG in a wide variety of human neoplasms including those of the endometrium. Strong expression of mutant P53 protein in endometrial cancer has been associated with advanced stage and high-grade tumors. BCL2 is an anti-apoptotic protein and preserves cells from apoptosis by regulating mitochondrial membrane function. BCL2-associated X protein (BAX) encodes pro-apoptotic proteins that are responsible for inducing cell death. An excess of BAX expression results in cell death, whereas overload BCL2 expression inhibits apoptosis. It has been suggested that dysfunction of the P53/BCL2/BAX apoptosis signaling pathway plays a role in tumorigenesis and tumor progression (Argiris et al., 2006[1]; Vaskivuo et al., 2000[35]).

In the present study, we investigated the potential anti-tumor activity of perifosine, vitamin D and combinations of both on the human endometrial cancer cell line (HEC-1A). 

## Materials and Methods

### Reagents

The perifosine (Sigma-Aldrich, Merck, Istanbul, Turkey) was dissolved in DMSO (concentration of the stock solution = 10 mM). Calcitriol (Cayman Chemical, Michigan, USA) was reconstituted in 100 % ethanol and stored at −20 °C. 

### Cell culture

HEC-1A cells were obtained from American Type Culture Collection (ATCC^® ^HTB112^™^, Manassas, VA, USA). HEC-1A cells were cultured in McCoy’s 5A Modified Medium (Thermo Fisher Scientific, Inc., Waltham, MA, USA) supplemented with 10 % fetal bovine serum, penicillin/ streptomycin and 1 % amphotericine-B. Cell culture was performed at 37 °C in 5 % CO_2_ and in a humidified (95 % RH) incubator. HEC-1A cells were incubated with increasing concentrations of perifosine (10 μM and 30 μM), vitamin D (50 nM and 200 nM) and combinations of both (10 μM + 50 nM, 10 μM + 200 nM, 30 μM + 50 nM and 30 μM + 200 nM) for 48 h and 72 h. HEC-1A cells without treatment were used as control. 

### xCELLigence real-time cell analysis

Cell proliferation and drug-mediated cytotoxicity were monitored real-time through the xCELLigence Real-Time Cell Analyser (RTCA) Dual Plate (DP) (Roche Diagnostics GmbH, Penzberg, Germany) system by measuring cell-to-electrode responses of HEC-1A cells seeded in E-plates well (2x10^4^ cells/well). For the proliferation assay, after the initial 24 h of proliferation, perifosine (10 μM, 30 μM, and 50 μM), vitamin D (50 nM and 200 nM) and combinations of both (10 μM + 50 nM, 10 μM + 200 nM, 30 μM + 50 nM, 30 μM + 200 nM, 50 μM + 50 nM, 50 μM + 200 nM) were added to each well and were monitored real-time for 72 h. Cell index (CI) value was automatically calculated for each E-plate well by RTCA Software (1.2.1).

### RNA isolation and quantitative real-time reverse transcription polymerase chain reaction (RT-qPCR)

Total RNA from HEC-1A cells was isolated using the high pure RNA isolation kit (cat. no. 1828665; Roche Life Science, Mannheim, Germany) according to the manufacturer’s instructions. Afterwards, cDNA was synthesized from 10 μg of total RNA using cDNA kit (cat. no. 4896866; Roche Life Science, Mannheim, Germany) and was stored at –20 °C until real-time reaction.

Gene expression analysis of BCL2, BAX, and P53 was performed using Lightcycler 480 II (Roche Life Science, Mannheim, Germany). Amplification reactions were set up in a reaction volume of 20 μl using the LightCycler 480 PCR Master Mix (cat. no. 04707494001; Roche Life Science, Mannheim, Germany). The reaction conditions after incubation at 95 °C for 10 seconds, denaturation at 95 °C for 10 minutes followed by 45 cycles of 60 °C for 30 seconds, 72 °C for 1 minute. The relative expression of genes was calculated by the comparative 2^–∆∆Ct ^method using beta-actin (ACTB) RNA levels as internal control (Livak and Schmittgen, 2001[23]). PCR primers and TaqMan probes were synthesized and preoptimized. The primer sequences of each gene are listed in Table 1(Tab. 1). Probe sequences were commercially reserved by company.

### Detection of apoptosis by flow cytometry

Apoptosis was evaluated by flow cytometry using BioLegend's APC Annexin V Apoptosis Detection Kit with propidium iodide (PI) (cat. no. 640932 BioLegend, San Diego, CA) following the manufacturer's instructions. Prepared cells were analyzed on the BD FACSAria™ III flow cytometer (BD Biosciences, Bedford, MA, USA) using FACSDiva Software. A total of 10,000 events per condition was recorded. Live cells are negative for both Annexin V APC and PI. Early apoptotic cells are Annexin V APC positive and PI negative. Late apoptotic/necrotic cells are positive for both Annexin V APC and PI.

### Transmission electron microscopy

HEC-1A cells were cultured in the McCoy’s 5A culture medium, perifosine, vitamin D or combinations of both for 72 h at 37°C. Cells were harvested, washed twice with PBS, and fixed with 2.5 % glutaraldehyde in 0.1 mol/L PBS (pH 7.4) at room temperature for 90 min. After fixation, samples were postfixed in 1 % osmium tetroxide, dehydrated in a graded series of ethyl alcohol and embedded in Embed-812 epoxy resin (cat. no. 14120; Electron Microscopy Sciences, Hatfield, PA). The blocks were cut into ultrathin sections (70 nM). Then, the sections were contrasted with uranyl acetate and lead citrate. The cell ultrastructure analysis was performed using a JEOL JEM-1011 microscope (JEOL Ltd., Tokyo, Japan).

### Statistical analysis

The statistical calculations were performed using STATISTICA Version 13.3 software. All *in vitro* studies were carried out in triplicate and results were expressed as means ± SD. The repeated measures ANOVA test was used as multiple comparison test to compare the statistical differences between group and time interactions. Statistical significance between groups was evaluated with Tukey-HSD for post-hoc multiple comparisons. P<0.05 was considered statistically significant.

## Results

### Anti-proliferative effects in real time cell analysis system

**Figure 1:**
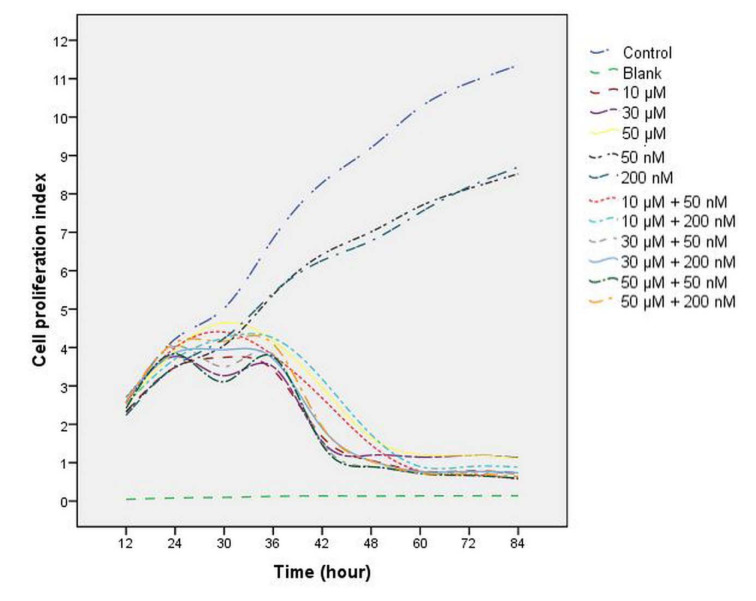
The effect of perifosine (10 μM, 30 μM, and 50 μM), vitamin D (50 nM and 200 nM) and combinations of both (10 μM + 50 nM, 10 μM + 200 nM, 30 μM + 50 nM, 30 μM + 200 nM, 50 μM + 50 nM, 50 μM + 200 nM) on HEC-1A cell proliferation. Cell proliferation index was examined for 84 h using xCELLigence RTCA.

The data demonstrated that after exposure to perifosine, vitamin D and combinations of both, the cell proliferation index value was reduced in a time-dependent manner compared with the control group (Figure 1(Fig. 1)). A difference in a statistical significance was not found between groups after the treatment at 24 h (all comparisons P>0.05), (Table 2(Tab. 2), Figure 1(Fig. 1)). A significant decrease in cell proliferation was observed in perifosine groups (10 μM, 30 μM, and 50 μM), vitamin D groups (50 nM and 200 nM) and combination groups (10 μM + 50 nM, 10 μM + 200 nM, 30 μM + 50 nM, 30 μM + 200 nM, 50 μM + 50 nM, 50 μM + 200 nM) when compared to control group after the treatment at 36 h, 48 h, and 72 h (all comparisons p<0.05), (Table 2(Tab. 2), Figure 1(Fig. 1)). The cell proliferation was decreased significantly in perifosine groups and combination groups compared with 50 nM and 200 nM vitamin D groups after the treatment at 36 h, 48 h, and 72 h (all comparisons p<0.05) (Table 2(Tab. 2), Figure 1(Fig. 1)). The IC_50_ value of perifosine was calculated as 30 μM. 

### The effect of perifosine, vitamin D and combinations of both on the expression levels of BCL2, BAX and P53

**Figure 2:**
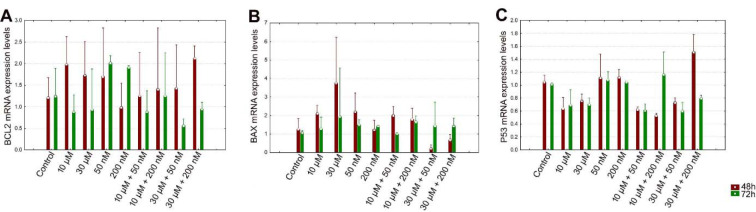
Evaluation of the BCL2 (A), BAX (B), P53 (C) mRNA expression levels of perifosine, vitamin D and combinations of both for 48 h and 72 h

The level of BCL2 mRNA expression was decreased in perifosine groups, 10 μM + 50 nM and 30 μM + 50 nM combination groups compared to 50 nM vitamin D group significantly for 72 h (all comparisons p<0.05), (Table 3(Tab. 3), Figure 2A(Fig. 2)).

After 48 h incubation period, there was a significant increase in the level of BAX mRNA expression just in 30 μM perifosine group compared to control (p<0.05), (Table 3(Tab. 3), Figure 2B(Fig. 2)). 

After 48 h incubation period, the level of P53 mRNA expression was decreased in perifosine groups, 10 μM + 50 nM, 10 μM + 200 nM, and 30 μM + 50 nM combination groups and increased in 30 μM + 200 nM combination group compared to control significantly (all comparisons p<0.05), (Table 3(Tab. 3), Figure 2C(Fig. 2)). After 72 h incubation period, the level of P53 mRNA expression was increased in vitamin D groups and 10 μM + 200 nM combination group compared to perifosine groups significantly (all comparisons p<0.05), (Table 3(Tab. 3), Figure 2C(Fig. 2)).

### Perifosine and vitamin D induces cell apoptosis in HEC-1A cells

**Figure 3:**
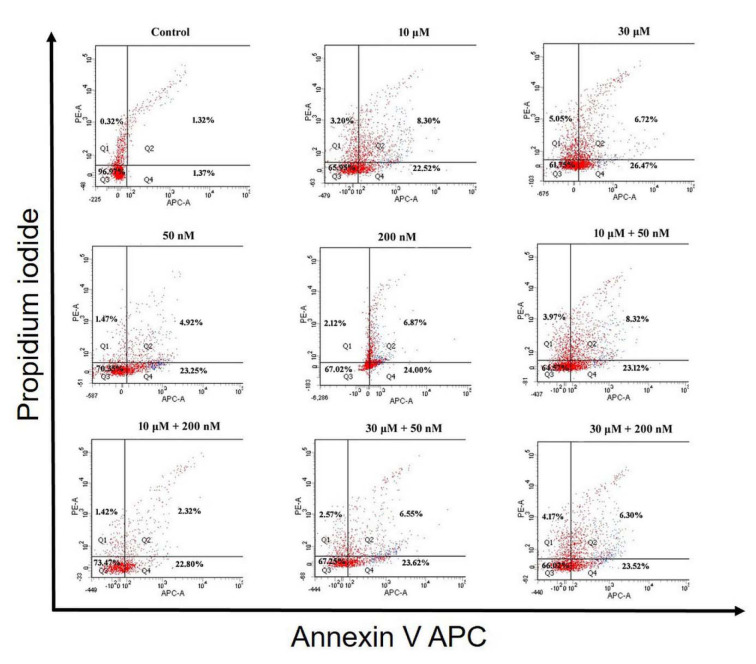
Apoptosis analysis of HEC-1A cells to detect live cells (Q3), early apoptotic cells (Q4), late apoptotic cells (Q2) and necrotic cells (Q1) by flow cytometry, using Annexin V APC/PI apoptosis assay after incubation with perifosine (10 μM and 30 μM), vitamin D (50 nM and 200 nM) and combinations of both (10 μM + 50 nM, 10 μM + 200 nM, 30 μM + 50 nM, 30 μM + 200 nM) for 48 h

According to the results that were obtained after 48 h incubation period in HEC-1A cells, there was a significant decrease in the percentage of live cells in other groups compared to control (all comparisons p<0.05), (Table 4(Tab. 4), Figure 3(Fig. 3) and 5A). The mean percentage of early apoptotic cells in HEC-1A cells was significantly increased in other groups compared to control (all comparisons p<0.05), (Figure 3(Fig. 3) and 5A). The mean percentage of late apoptotic cells has increased in 10 μM perifosine and 10 μM + 50 nM combination group compared to control significantly (all comparisons p<0.05), (Figure 3(Fig. 3) and 5A). 

**Figure 4:**
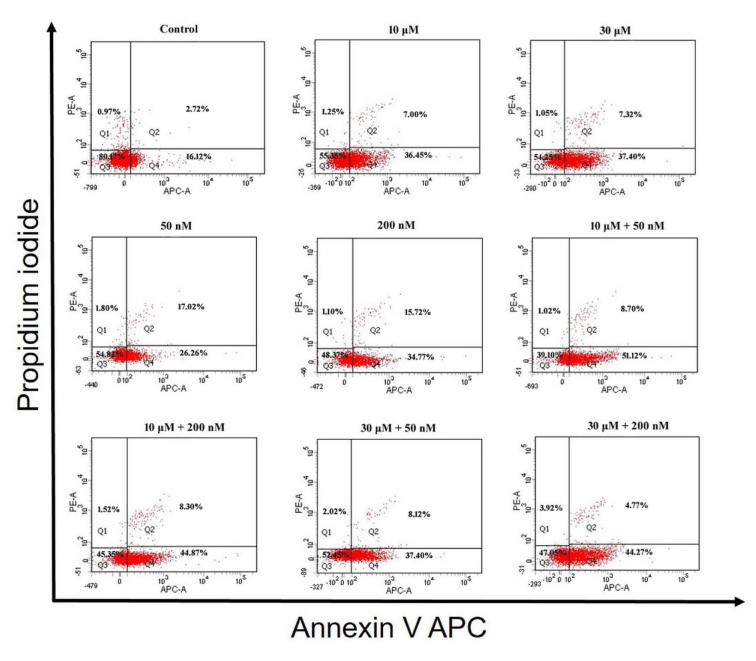
Apoptosis analysis of HEC-1A cells to detect live cells (Q3), early apoptotic cells (Q4), late apoptotic cells (Q2) and necrotic cells (Q1) by flow cytometry, using Annexin V APC/PI apoptosis assay after incubation with perifosine (10 μM and 30 μM), vitamin D (50 nM and 200 nM) and combinations of both (10 μM + 50 nM, 10 μM + 200 nM, 30 μM + 50 nM, 30 μM + 200 nM) for 72 h

**Figure 5:**
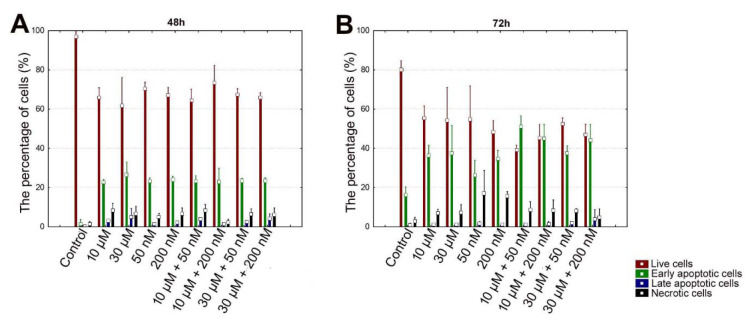
Graph of apoptosis analysis of HEC-1A cells to detect live cells, early apoptotic cells, late apoptotic cells and necrotic cells by flow cytometry after incubation with perifosine (10 μM and 30 μM), vitamin D (50 nM and 200 nM) and combinations of both (10 μM + 50 nM, 10 μM + 200 nM, 30 μM + 50 nM, 30 μM + 200 nM) for 48 h (A) and 72 h (B)

According to the results that were obtained after 72 h incubation in HEC-1A cell, there was a significant decrease in the percentage of live cells in other groups compared to control (all comparisons p<0.05), (Table 5(Tab. 5), Figure 4(Fig. 4) and 5B(Fig. 5)). When comparing perifosine groups, 50 nM vitamin D group, we have found a decrement in mean percentage of live cells in 10 μM + 50 nM combination group (p<0.05), (Figure 4(Fig. 4) and 5B(Fig. 5)). The mean percentage of early apoptotic cells in HEC-1A cells was significantly increased in other groups compared to control (all comparisons p<0.05), (Figure 4(Fig. 4) and 5B(Fig. 5)). The mean percentage of early apoptotic cells was significantly increased in perifosine groups and all of combination groups compared to 50 nM vitamin D group (all comparisons p<0.05) (Figure 4(Fig. 4) and 5B(Fig. 5)). The same increment was found in 10 μM + 50 nM, 10 μM + 200 nM, and 30 μM + 200 nM combination groups compared to 200 nM vitamin D group significantly (all comparisons p<0.05) (Figure 4(Fig. 4) and 5B(Fig. 5)). The mean percentage of late apoptotic cells in HEC-1A cells has increased in 50 nM and 200 nM vitamin D groups compared to control and perifosine groups significantly (all comparisons p<0.05) (Figure 4(Fig. 4) and 5B(Fig. 5)).

### Ultra-structural alterations

**Figure 6:**
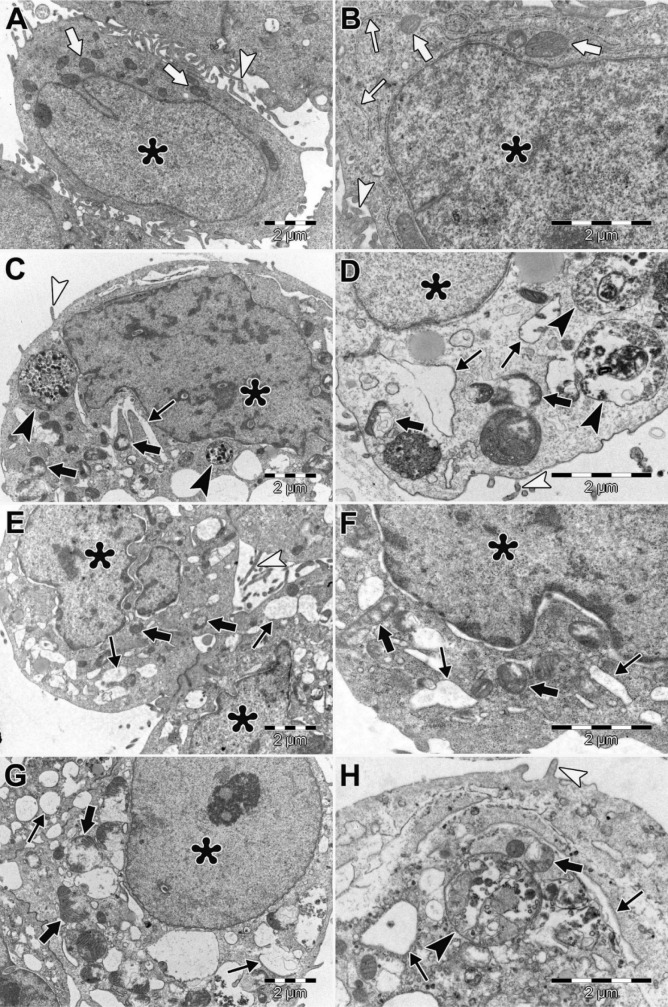
Ultrastructural morphology of HEC-1A cells. HEC-1A cells of control, nucleus (asterisks), normal mitochondria (thick arrows) and normal ER (thin arrows), microvilli (arrowheads), (A, B). HEC-1A cells treated with perifosine (C, D). HEC-1A cells treated with vitamin D (E, F). HEC-1A cells treated with combinations of perifosine and vitamin D (G, H). Nucleus (asterisks), degenerated mitochondria (thick arrows), swollen and fused ER (thin arrows), autophagic vacuoles (black arrowheads), microvilli (white arrowheads). (A, C, E, G) X10000; (B, D, F, H) X20000. Uranyl acetate-lead citrate

**Figure 7:**
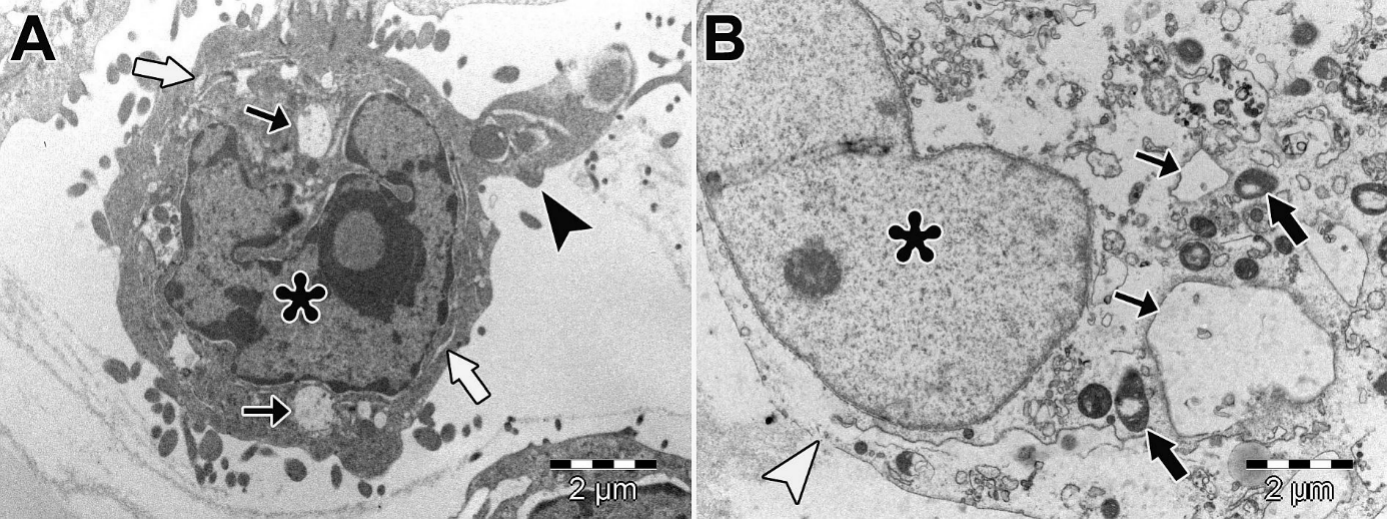
Ultrastructural morphology of HEC-1A cells. Apoptotic HEC-1A cell (A). Necrotic HEC-1A cell (B). Nucleus (asterisks), degenerated mitochondria (black thick arrows), swollen ER (white thick arrows), large vacuoles (thin arrows), blebbing of apoptotic bodies (black arrowhead), ruptured plasma membrane (white arrowheads). X 10000. Uranyl acetate-lead citrate

In HEC-1A cells of control, ultrastructure of cell membrane cytoplasmic organelles, and nucleus was normal (Figure 6A and 6B(Fig. 6)). We observed large autophagic vacuoles, empty vacuoles with varying size, swollen endoplasmic reticulum (ER) and degenerated mitochondria in HEC-1A cells treated with perifosine (10 μM and 30 μM) (Figure 6C and 6D(Fig. 6)). We found swollen and fused ER, degenerated mitochondria and increased perinuclear space in HEC-1A cells treated with vitamin D (50 nM and 200 nM), (Figure 6E and 6F(Fig. 6)). In both groups, we showed some apoptotic cells that include intact nuclear membrane, plasma membrane blebbing, chromatin marginalization, nuclear fragmentation (Figure 6G and 6H(Fig. 6)). We detected large autophagic vacuoles, swollen ER and degenerated mitochondria and apoptotic cells in HEC-1A cells treated with combinations of perifosine and vitamin D (Figure 7A(Fig. 7)). Many necrotic cells were also observed in combination groups (Figure 7B(Fig. 7)).

## Discussion

Endometrial cancer constitutes 4-6 % of cancers seen in women (Parkin et al., 2002[29]). Treatment of advanced or repeated endometrial cancer remains an essential problem; for this reason, the effects of various agents are being investigated intensively. Studies have shown that perifosine and vitamin D have anti-proliferative effects on different cancer cells, but the effects of combinations of both on the endometrial cancer cell line are still unknown (Elrod et al., 2007[8]; Getzenberg et al., 1997[13]; Hershberger et al., 2001[16]). Hence, we aimed to investigate the possible anti-proliferative and apoptotic changes in the HEC-1A cells that were treated with perifosine, vitamin D and combinations of both.

Perifosine is a synthetic alkylphospholipid, which inhibits AKT activation (Kondapaka et al., 2003[20]). AKT plays a key role in the regulation of tumor-related cellular processes such as cell growth, cell cycle progression, cell migration, epithelial-mesenchymal transition and angiogenesis (Cheng et al., 2005[5]). Therefore, AKT inhibitors have become a promising agent for cancer treatment nowadays. Many studies performed with different types of cancer revealed the anti-tumor activity of perifosine at a dose range of 1-50 μM (Elrod et al., 2007[8]; Hideshima et al., 2006[17]). Previous studies demonstrated anti-carcinogenic effects for vitamin D compounds (Mondul et al., 2017[27]). Studies have shown that vitamin D has anti-proliferative roles through a variety of mechanisms including cell cycle arrest, apoptosis, and induction of differentiation at the dose range of 1-400 nM in different types of cancer (Getzenberg et al., 1997[13]; Hershberger et al., 2001[16]; Wigington et al., 2004[39]). In addition, these studies demonstrated that the combination of vitamin D with other chemotherapeutic agents might lead to inhibition of tumor growth (Axanova et al., 2010[2]; Ma et al., 2008[24]). In this study, for cytotoxicity response of HEC-1A cells, while a statistical difference was not found significantly between other groups compared to control group after the treatment at 24 h, there was a significant decrease in cell proliferation for perifosine (10 μM, 30 μM and 50 μM), vitamin D (50 nM and 200 nM) and combination groups (10 μM + 50 nM, 10 μM + 200 nM, 30 μM + 50 nM, 30 μM + 200 nM, 50 μM + 50 nM, 50 μM + 200 nM) compared to control group at 36 h. It was determined that cell proliferation decreased in all perifosine, vitamin D and combination groups according to control group at 48 h and 72 h. Engel et al. indicated that perifosine inhibited the cell proliferation by blocking AKT pathway through inhibition of AKT phosphorylation in Ishikawa (1.25 μM and 7 μM) and HEC-1A (6 μM and 25 μM) cells (Engel et al., 2008[9]). Axanova et al. showed that AKT inhibitors (API-2 and GSK690693) combined with vitamin D (1 nM, 10 nM, 100 nM) synergistically inhibit the growth of prostate cancer cells (Axanova et al., 2010[2]). In our study, in accordance with these results, a combination of perifosine and vitamin D inhibited the proliferation of HEC-1A cells. Furthermore, we demonstrated that the administration of vitamin D alone has an inhibiting effect on the proliferation of HEC-1A cells in a time-dependent manner, but that inhibition was more efficient when combined with perifosine. Therefore, we assumed that the combination of vitamin D with perifosine showed an anti-proliferative effect by synergistic impact and could be a new alternative treatment in clinical approach for endometrial cancer.

The P53 transcription factor was expressed depending on the presence of DNA damage in the cell to stop the cell cycle in G1 phase and plays a central role in the activation of DNA repair mechanisms (Campomenosi et al., 2001[4]). In cases where the DNA cannot be repaired, the P53 gene induces apoptosis by increasing BAX expression, decreasing of BCL2 expression, or stimulating death receptors such as Fas and tumor necrosis factor (TNF) receptors (Vousden and Lu, 2002[38]). Elrod et al. indicated that perifosine (8 to 15 μmol/L) induced expression of death receptor 5 (DR5) in human non–small cell lung cancer (NSCLC) cell lines (H157 and A549), decreased FLICE-inhibiting protein (c-FLIP) and BAX expression levels, but had a limited modulatory effect on BCL2, Bcl-XL and p53 upregulated modulator of apoptosis (PUMA) levels (Elrod et al., 2007[8]). In this study, we examined the BCL2, BAX and P53 mRNA expression levels to reveal the possible apoptotic mechanisms. In our study, there was no significant difference on BCL2 mRNA expression level between groups for 48 h and 72 h. The level of BAX mRNA expression was increased only in 30 μM perifosine group for 48 h. We indicated the effect of perifosine on BCL2/BAX pathway for 30 μM perifosine at 48 h. On the other hand, Fei et al. showed that perifosine caused activation for caspase-3, caspase-9 and Poly ADP Ribose Polymerase (caspase downstream effector, PARP) in human hepatocellular carcinoma cells (HepG2), but did not affect P53 and BCL2 levels (Fei et al., 2010[10]). In this study, we found that P53 mRNA expression level was significantly higher in the 30 μM + 200 nM combination group at 48 h and in the 10 μM + 200 nM combination group at 72 h compare to the other groups. According to this, we assumed that perifosine and vitamin D combination could induce apoptosis in HEC-1A cells by increasing the P53 mRNA expression level by synergistic effect. 

The clinical efficacy of chemotherapeutic drugs that is used in the treatment of cancer is associated with the formation of effective cell cycle arrest in cancer cells and increased apoptotic activation (Gerl and Vaux, 2005[12]; Huang et al., 2017[18]). In a study, they indicated that the combination of perifosine and histone deacetylase inhibitors (HDACI, sodium butyrate, suberosilanilide hydroxamic acid and tricostatin) was reported to increase apoptosis in human leukemia cells (U937, HL-60 and Jurkat cells) (Rahmani et al., 2005[31]). Pistor et al. showed that methylprednisolone (2.5 mM), vitamin D (100 nM) and MK-2206 (AKT inhibitor, 2 µM) have a synergistic effect on apoptosis of steroid-resistant T-cell acute lymphoblastic leukemia (T-ALL) cells (Pistor et al., 2018[30]). In this study, it was found that the mean percentage of apoptotic cells increased in all perifosine+vitamin D combination groups at 48 h and 72 h hours compared to control, whereas 10 μM + 50 nM combination group caused apoptosis more than 50 % of HEC-1A cells at 72 h. In addition, the mean percentage of apoptotic cells was higher in the combination groups (10 μM + 50 nM, 10 μM + 200 nM) than in the groups treated with perifosine and vitamin D alone. According to these results, we assumed that the combination treatment increased apoptosis more strenuously rather than single agent usage. However we needed more studies to demonstrate clinical efficiency of perifosine and vitamin D combination therapy.

In this study, apoptotic and non-apoptotic cell death was observed in HEC-1A cells treated with perifosine, vitamin D and combinations of both by TEM examinations. Both autophagic vacuoles and apoptotic bodies were detected in HEC-1A cells treated with perifosine suggesting that perifosine induced cell apoptotic and autophagic cell death in HEC-1A. Autophagic activity is mostly controlled by PI3K/AKT/mTOR signaling pathway (Sarbassov et al., 2005[33]). Previous studies indicated that perifosine induces autophagy by inhibiting mTOR signaling while initiates apoptosis in human lung cancer cells (Elrod et al., 2007[8]; Fu et al., 2009[11]). We observed paraptosis-like cell death involved degenerated mitochondria, swollen, fused ER and apoptotic cell death in HEC-1A cells treated with vitamin D. Similarly, Haddur et al., suggested an association between ER stress and vitamin D signaling in a breast cancer cell (MCF7, MDA-MB-231) (Haddur et al., 2015[14]). Mathiasen et al. reported that vitamin D analogues (calcitriol and EB 1089) induced caspase-independent cell death in breast cancer cells (MCF-7) while inducing apoptosis-like ultrastructural changes characterized by condensation in the cytoplasm and chromatin content of the cells. Moreover, they demonstrated dilate ER cisternas that were caused by ER stress (Mathiasen et al., 2002[26]). According to these results, we supposed that vitamin D caused cell death through the independent caspase pathway in HEC-1A cells. In relation with these information, we need to carry out more researches to reveal the significance of relation between vitamin D, ER stress and paraptosis-like cell death. Cirstea et al. indicated the apoptotic and autophagic cell death in multiple myelom cells that were treated with perifosine and rapamycin combination as ultrastructural (Cirstea et al., 2010[6]). We observed that the combination of perifosine and vitamin D treatment could trigger multiple cell death mechanisms such as apoptosis, paraptosis-like cell death and autophagic cell death. Overall, we assumed that the combination treatment was more efficient rather than using these agents separately. 

Consequently, our results exhibited that perifosine, vitamin D and combination of them may cause inhibition of proliferation in endometrial cancer cells and activates apoptotic and non-apoptotic cell death mechanism. In endometrial cancer cells, 30 μM + 200 nM combination group at 48 h and 10 μM + 200 nM combination group at 72 h have more effective apoptotic effect than perifosine and vitamin D treatment alone. Therefore, it was thought that the combination of both drugs could be an alternative and effective treatment option for cancer chemotherapy by activating autophagic and paraptotic cell death pathways besides apoptotic pathways. However, molecular mechanisms of perifosine+ vitamin D combinations that lead to increased cell death in endometrial cancer cells should be demonstrated clearly and new clinical studies related to them should be performed.

## Acknowledgements

This study was supported by the Mersin University Department of Scientific Research Projects under Grant 2016-2-TP3-1888.

## Conflict of interest

The authors declare that they have no conflict of interest.

## Figures and Tables

**Table 1:**
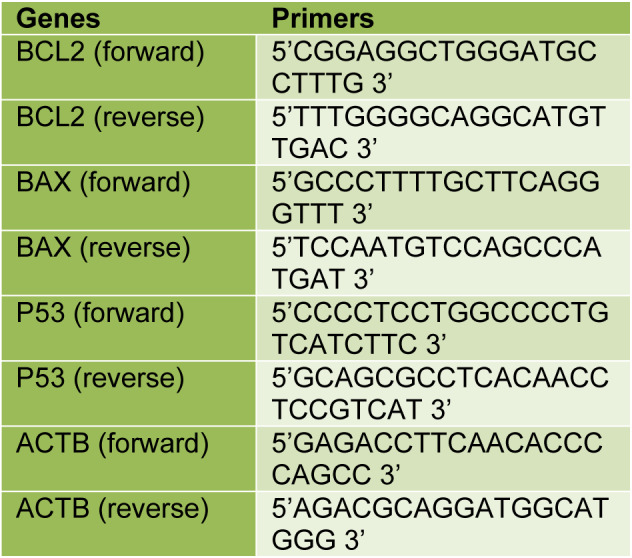
The primer sequences of each gene

**Table 2:**
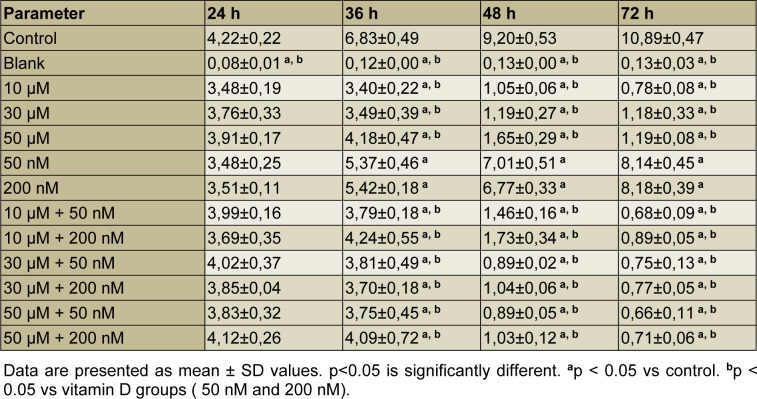
Cell proliferation index of HEC-1A cells treatment with the perifosine (10 μM, 30 μM, and 50 μM), vitamin D (50 nM and 200 nM) and combinations of both (10 μM + 50 nM, 10 μM + 200 nM, 30 μM + 50 nM, 30 μM + 200 nM, 50 μM + 50 nM, 50 μM + 200 nM)

**Table 3:**
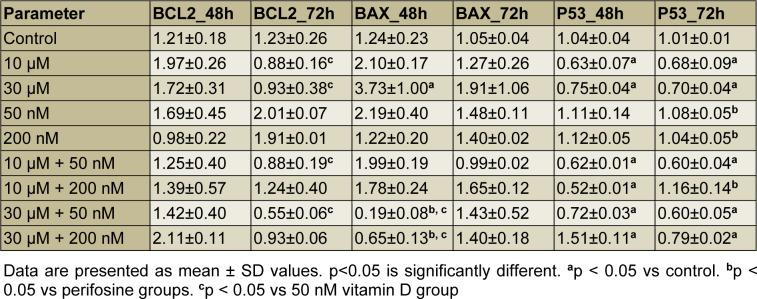
The levels of BCL2, BAX and P53 mRNA expression for 48 h and 72 h

**Table 4:**
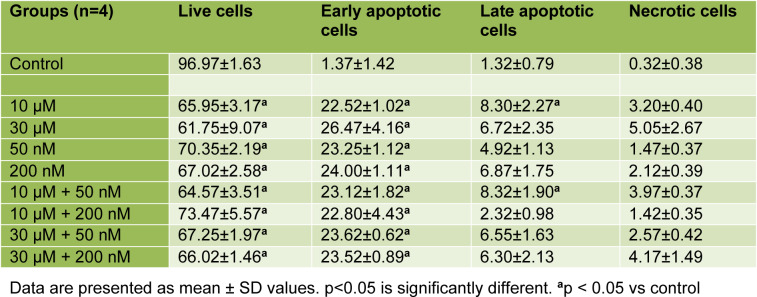
Mean values of live cells, early apoptotic cells, late apoptotic cells and necrotic cells for 48 h

**Table 5:**
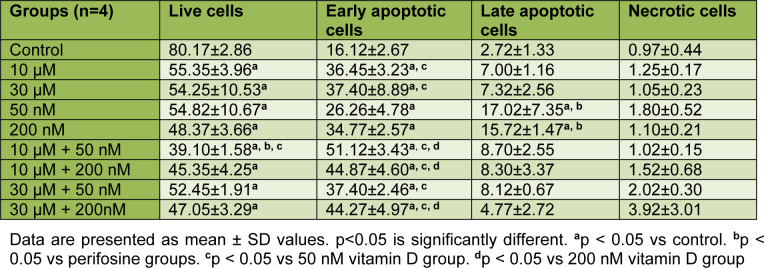
Mean values of live cells, early apoptotic cells, late apoptotic cells and necrotic cells for 72 h
